# Pathologically confirmed women's breast cancer: A descriptive study of Tunisian and Algerian series

**DOI:** 10.1002/cnr2.1818

**Published:** 2023-04-24

**Authors:** Farah Sassi, Meriem Ben Rekaya, Ayed Belarbi, Dalia Chilla, Nada Mansouri, Leila Achouri, Essia Saied, Reda Kassa, Linda Belhaj Kacem, Manel Ouezani, Nadjiba Debabeche, Fatima Rebhi, Soumaya Rammeh

**Affiliations:** ^1^ Department of Pathology, Charles Nicolle Hospital Tunis, UR17ES15, Faculté de Médecine de Tunis Université de Tunis El Manar Tunis Tunisia; ^2^ Department of Pathology, Douéra Hospital, Laboratoire de Recherche polymorphisme génétique No. 18 Université d'Alger 1 Alger Centre Algeria; ^3^ Department of Pathology Military Hospital Tunis Tunisia; ^4^ Department of Surgical Oncology Regional Hospital of Jendouba Jendouba Tunisia

**Keywords:** Algeria, breast cancer, immunohistochemistry, molecular breast cancer subtypes, pathology, Tunisia

## Abstract

**Background:**

Breast cancer (BC) is the most frequent malignancy among women in Tunisia and Algeria. Clinical and pathological characteristics of this cancer among these populations are not widely reported. The aim of the study was to report clinical and pathological characteristics of women's BC in a Tunisian and Algerian series.

**Methods:**

Pathologically confirmed 1089 BCs were gathered in the pathology departments of three Northern Tunisian hospitals: Tunis military, Charles Nicolle and Jendouba and in the pathology department of Alger Douera hospital between January 2015 and December 2020. Clinical and pathological findings of the two series: age, tumor size, histological type, grading according to Scarff‐Bloom Richardson grading system, lymph node status at the time of diagnosis in axillary lymphadenectomy specimens and the immunohistochemical expression of estrogen and progesterone receptors (ER/PR), HER2 and Ki‐67, were collected from the pathological reports.

**Results:**

The median age at diagnosis was 50 and 48 years in Tunisian and Algerian series, respectively (*p* = 0.016). The diagnosis of BC was made on surgical specimens (lumpectomy or mastectomy) in 373/491 (76%) cases of the Tunisian series and in 225/598 (37.6%) cases of the Algerian one. Median tumor size was 2.8 cm and 2.5 cm in Algerian and Tunisian series, respectively (*p* = 0.252). Invasive BCs not otherwise specified was observed in 440/481 (91.5%) BCs in Tunisian series and in 519/586 (88.6%) BCs in Algerian series. Axillary lymph node positive tumors were observed in 64.6% and 58.8% of Tunisian and Algerian women, respectively (*p* = 0.926). BCs were ER positive in 311/385 (80.8%) and 486/571 (85.1%) cases and HER2 positive in 86/283 (30.4%) and 60/385 (15.6%) cases of Tunisian and Algerian series, respectively.

**Conclusions:**

In Tunisia and Algeria, BC has poor prognostic factors with large tumor sizes and high rates of lymph nodes involvement at diagnosis.

## 1 INTRODUCTION

With 2.2 million new cases and 68 499 deaths in 2020, breast cancer (BC) is the most frequent malignancy in women and the first major cause of cancer death in women globally.^1^ The highest incidence rates (>80 per 100 000 females) are observed in Australia and New Zealand, Western Europe, Northern America, and Northern Europe. The lowest rates (40 per 100 000 females) are observed in Central America, Eastern and Middle Africa, and South‐Central Asia. The highest mortality rates (>20 per 100 000 females) were found in Melanesia, Western Africa and Micronesia/Polynesia, while rates in most other world regions range between 10 and 15 per 100 000 females.^2^ These rates reflect the availability of mammography, thus, the detection of BCs at an early stage in high‐income countries. BC is often discovered at a later stage in low‐ and middle‐income countries, which partly explains the high mortality rates in these regions.^3^

In Northern Africa, the Global Cancer Observatorygco database estimates that there were 57 128 new female BC cases and 21 524 related deaths in 2020. In Tunisia, BC is a major public health issue, accounting for 34.5% of all female malignancies with 3092 annual diagnosed cases in 2020.^1^ In Algeria, BC is also a leading cause of cancer, accounting for 40.3% of all female malignancies, with 12 536 new cases diagnosed in 2020. Its incidence in Tunisia and Algeria has been increasing at an alarming rate for about 25 years. The data from the Tunisian and Algerian registries illustrate this real and regular increase.^4^, ^5^

According to World Health Organization (WHO) classification of breast tumors, BC has a broad spectrum of histological patterns.^6^ BCs with distinct histological patterns are classified as a special tumor type, such as lobular, mucinous, or tubular carcinomas etc… Invasive BC of non‐specific subtype is the term used to describe BCs lacking sufficient characteristics to achieve classification as a specific histological type.^6^

With the development and implementation of genomic and expression profiling analyses, the Cancer Genome Atlas (TCGA) Network has helped establish refined subtypes of BC through extensive profiling of protein levels, mRNA level, and DNA.^7^ The molecular classification changed the paradigm of BC treatment. It includes four subtypes: “luminal A,” “luminal B,” “HER2‐positive,” and “basal‐like.”^8^ Patients with luminal A BC have an excellent prognosis and gain clinically relevant benefits from endocrine therapy but not from chemotherapy. Patients with luminal B BC, however, may have a worse prognosis if treated alone with endocrine therapy due to endocrine resistance, but they may benefit from chemotherapy.^8^ HER2‐positive BCs are responsive to anti‐HER2 medications.^9^ Finally, chemotherapy is beneficial for patients with basal‐like BC.^10^

The frequencies of molecular subtypes vary worldwide. In Western series, luminal A subtype is predominant followed by luminal B, HER2, and basal‐like.^11^, ^12^, ^13^ Few studies of the clinicopathological features of BC in African countries have been conducted. The results of the published studies show some degree of divergence. In Sub‐Saharan Africa, BCs exhibit more aggressive features such as triple negative phenotype.^14^ Other studies indicate geographical variability in the distribution of BC molecular subtypes with the risk of triple negative BCs found to be lower in East Africa.^15^ In North African studies, luminal A is the predominant subtype.^16^, ^17^

Studies have shown that it is possible to reproduce this molecular classification by using immunohistochemical tests based on the expression of estrogen receptors (ER), progesterone receptors (PR), HER2, Ki‐67 and other biomarkers such as high and low molecular weight cytokeratins: CK5/6.^18^ Due to time and cost constraints, in the great majority of health care systems, surrogate molecular BC classification is largely based on immunohistochemical assessment of biomarkers. To discriminate between luminal A/B, HER2‐positive, and triple negative tumors, a panel encompassing ER, PR, HER2 and Ki‐67 might be employed.^6^

Currently, little is known about the clinical and pathological characteristics of BCs in North Africa, especially in Tunisia and Algeria.^16^, ^19^, ^20^ To our knowledge, no comparative study on BC clinicopathological characteristics among Tunisian and Algerian women has been published so far. To gain further insight into BC among the two populations, we studied the clinicopathological characteristics of BC series. This study is the first to present a large population‐based study on BC among Tunisian and Algerian women.

## 2 MATERIALS AND METHODS

### 2.1 Clinical and histological data

A retrospective and descriptive study was performed including all women's primitive BCs diagnosed between January 2015 and December 2020 in the pathology departments of three Northern Tunisian hospitals: Tunis military, Charles Nicolle and Jendouba and in the pathology department of Alger Douera hospital. Non‐primitive BCs and non‐carcinomatous breast tumors were not included. BCs with unavailable pathology reports were excluded from the study. Clinical and pathological characteristics were taken from pathological reports including: age classified into 6 subgroups <30, 31–40, 41–50, 51–60, 61–70 and >70, menopausal status, tumor size divided into <2 and ≥2 cm, histological type, grading according to Scarff‐Bloom Richardson (SBR) histological system and axillary lymph node status at the time of diagnosis in lymphadenectomy specimens.

### 2.2 Classification of breast cancer subtypes using immunohistochemical data

Four‐micrometer thick sections of Formalin‐Fixed Paraffin‐Embedded (FFPE) tissue was employed for the analysis of the expression of ER, PR, HER2, and Ki‐67 using immunohistochemistry (IHC). When the positive internal control was missing, an external one has been included in each immunostaining run. Antibodies clones used in Charles Nicolle and Jendouba's hospital were: ER (Clone SP1, rabbit monoclonal primary antibody, CONFIRM 790‐4325, Roche), PR (Clone 1E2, rabbit monoclonal primary antibody, CONFIRM 790‐2223, Roche), HER2 (Clone 4B5, rabbit monoclonal primary antibody, VENTANA 790‐4493, Roche), and Ki‐67 (Clone 30‐9, rabbit monoclonal primary antibody, CONFIRM 790‐4286, Roche) according to the manufacturer's guidelines. Antibodies clones used in Military hospitals were: ER (Clone 6F11, mouse monoclonal primary antibody, Leica Biosystems), PR (Clone 16, mouse monoclonal primary antibody, Leica Biosystems), HER2 (Clone CB11, mouse monoclonal primary antibody, Leica Biosystems), and Ki‐67 (Clone K2, mouse monoclonal primary antibody, Leica Biosystems), according to the manufacturer's guidelines. Antibodies clones used in Alger's hospital were ER (Clone EP1, mouse monoclonal primary antibody, DAKO), PR (Clone PgR636, mouse monoclonal primary antibody, DAKO), HER2 (Clone c‐erbB‐2, rabbit monoclonal primary antibody, DAKO), and Ki‐67 (Clone MIB‐1, mouse monoclonal primary antibody, DAKO).

ER and PR were considered positive if more than 10% of tumor cells showed nuclear positive immunostaining according to French recommendation.^21^ Human Epidermal growth factor receptor‐2 (HER2) was interpreted according to 2013 Saint Gallen consensus.

BCs were classified based on immunohistochemical profile as follows: Luminal A (ER+ and/or PR+/− and HER2−), luminal B (ER+ and/or PR+, Her2+), triple negative (ER−, PR− and HER2−) and HER2+ (ER−, PR− and HER2+). Index of proliferation (Ki‐67) was divided into two subgroups with a 20% cut‐off according to 2013 Saint Gallen consensus.^10^

### 2.3 Statistical analysis

Statistical analysis was performed by SPSS (version 26.0). Categorical data were summarized by frequencies and percentages. Continuous data regarding age and tumor size were presented as groups and medians. The assessment of associations between age, tumor size, histological subtype, histological grade, lymph node status, estrogen receptors, progesterone receptors, HER2, Ki‐67 cut‐off levels and molecular classification was performed using the *χ* ^2^ and Fisher's tests. *p* Values less or equivalent to 0.05 were considered significant.

## 3 RESULTS

From January 2015 to December 2020, 1089 pathologically confirmed BCs were enrolled: 598 BCs from Douera Hospital in Alger and 491 BCs from the three Northern Tunisian hospitals: 212 (43.2%), 154 (31.4%) and 125 (25.4%) from Jendouba, Military and Charles Nicolle hospitals, respectively. Median ages were 50 and 48 years in Tunisian and Algerian series, respectively (*p* = 0.016). The peak age of BCs was between 41 and 50 years in 162/491 (33.1%) Tunisian women and in 198/578 (34.2%) Algerian women. BCs diagnosed in women <40 years was observed among 87 (17.7%) Tunisian women and 148 (24.2%) Algerian women. BC occurred in 46.9% and 57.3% of premenopausal Tunisian and Algerian women, respectively.

**FIGURE 1:**
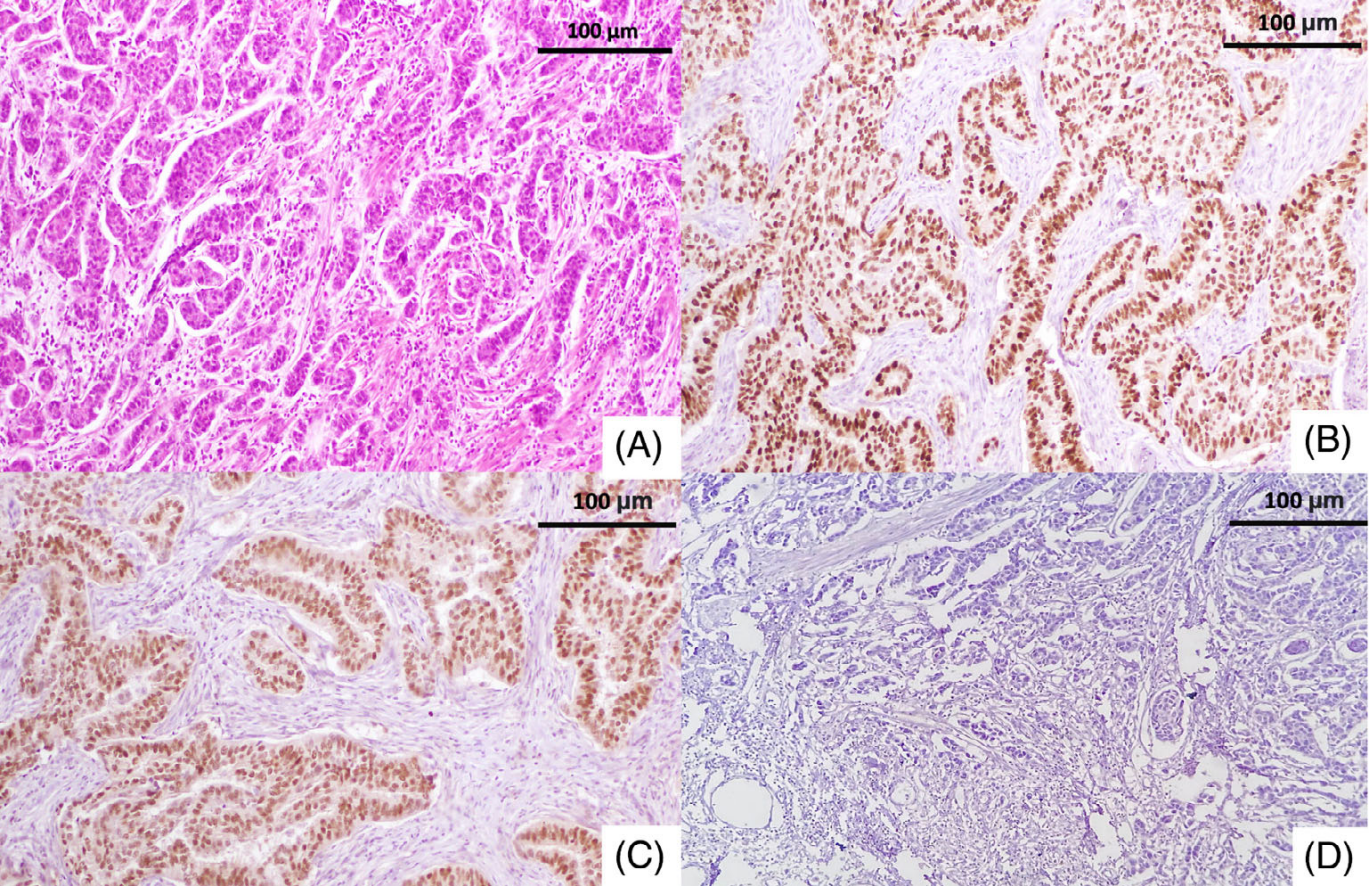
Luminal A molecular subtype A/Hematoxylin–eosin (HE) of an invasive carcinoma of no special type, original magnification 200×. Tumoral cells have enlarged nuclei, vesicular chromatin B/Diffuse and strong nuclear expression of estrogen receptors C/Diffuse and strong nuclear expression of progesteron receptors D/Lack of HER2 protein expression.

**FIGURE 2:**
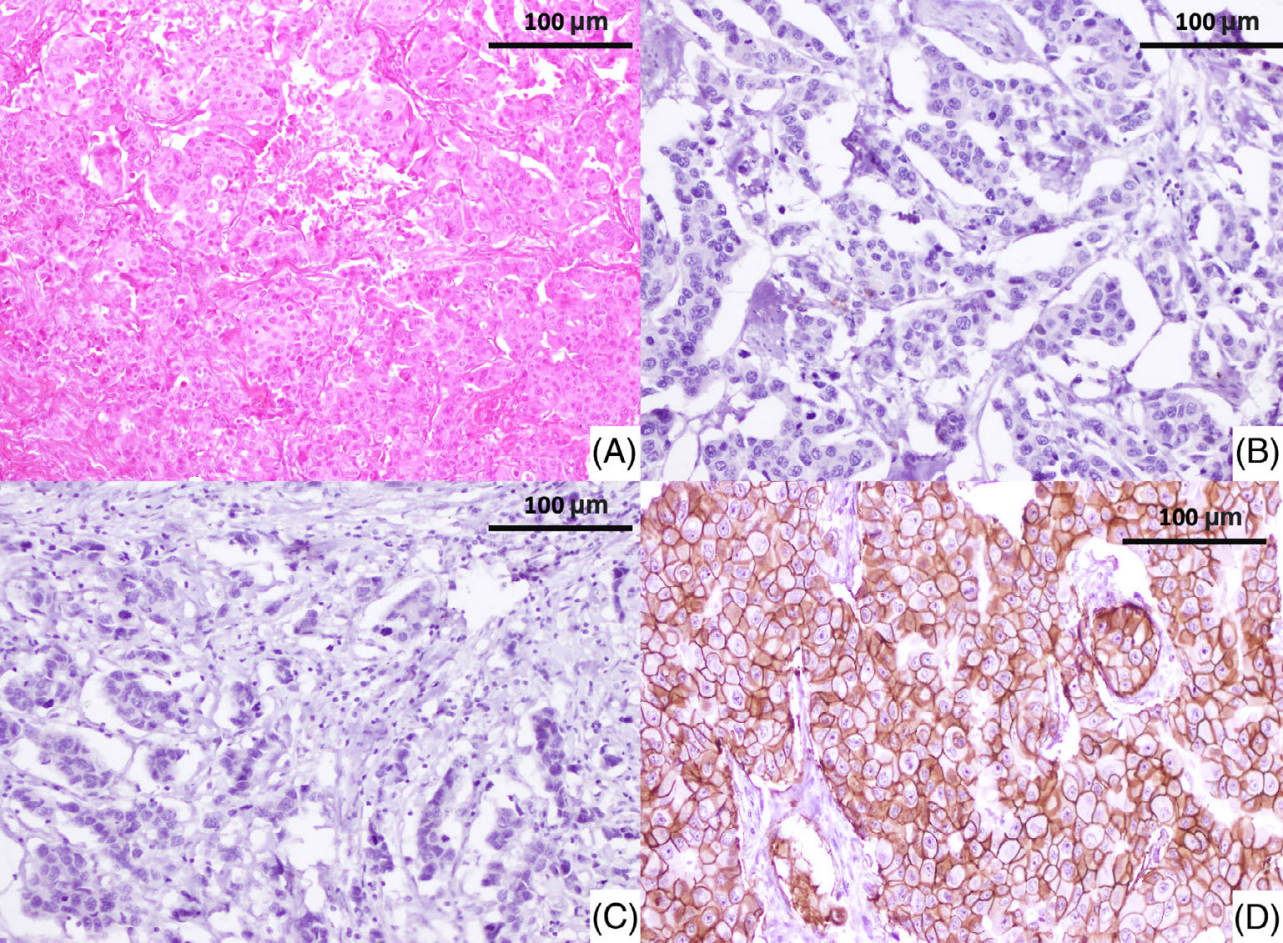
HER2+ molecular subtype A/Hematoxylin–eosin (HE) of an invasive breast carcinoma of no special type, original magnification 200×. Tumoral cells have enlarged nuclei, vesicular chromatin and prominent nuclei with mitotic figures B/Lack of estrogen receptor expression C/Lack of progesteron receptor expression D/HER2 protein overexpression (score 3+).

Pathological specimens were surgical (lumpectomy or mastectomy) in 373/491 (76%) cases of the Tunisian series and in 225/598 (37.6%) cases of the Algerian one. Median tumor size was 2.8 cm and 2.5 cm in Algerian and Tunisian series, respectively (*p* = 0.252). Invasive BCs not otherwise specified were diagnosed in 440/481 (91.5%) cases and 519/586 (88.6%) cases and invasive lobular BCs in 21/481 (4.4%) cases and 40/586 (6.8%) cases of Tunisian and Algerian series, respectively. According to SBR grading, BCs were grade I in 35/555 (6.3%) cases and 51/462 (11%) cases, grade II in 293/555 (52.8%) cases and 203/462 (44%) cases and grade III in 227/555 (40.9%) cases and 208/462 (45%) cases of Algerian and Tunisian series, respectively (*p* = 0.659). Axillary lymph nodes were positive in 144/223 (64.6%) cases and 120/204 (58.8%) cases of Tunisian and Algerian series, respectively (*p* = 0.926). In Tunisian and Algerian series, ER was positive in 311/385 (80.8%) BCs and 486/571 (85.1%) BCs, respectively (*p* = 0.186) and HER2 expression was positive in 86/283 (30.4%) BCs and 60/385 (15.6%) BCs, respectively (*p* = 0.123). Ki‐67 was ≥20% in 406/491 (82.7%) cases and in 407/544 (74.8%) cases of Tunisian and Algerian BCs, respectively (*p* = 0.426). In the Tunisian series, BCs were luminal A in 156/268 (58.3%) (Figure 1 **)** cases, luminal B in 59/268 (22%) cases, triple negative in 28/268 (10.4%) cases and HER2 positive in 25/268 (9.3%) cases **(**Figure 2 **)**. In the Algerian series, BCs were luminal A in 181/263 (68.8%) cases, luminal B in 22/263 (8.4%) cases, triple negative in 42/263 (16%) cases and HER2 positive in 18/263 (6.8%) cases. Differences between immunohistochemical subtyping of BCs in the two series were not significant (*p* = 0.673). Clinical and pathological characteristics of BCs in Tunisian and Algerian series are summarized in Table 1.

**TABLE 1:** Clinical and tumor characteristics of breast carcinomas among Tunisian and Algerian series.

Variables	Tunisian, *n* (%)	Algerian (%)	*p* Value
Total, *n*	491	598	
Age	491	578	
Range (years)	21–97	26–94	
Median	50	48	0.016
Age category			
<30	12 (2.4)	12 (2.1)	
31–40	75 (15.3)	128 (22.1)	
41–50	162 (33.1)	198 (34.2)	
51–60	119 (24.2)	127 (22)	
61–70	67 (13.6)	68 (11.8)	
>70	56 (11.4)	45 (7.8)	
Menopausal status	491	578	
Premenopausal	230 (46.9)	331 (57.3)	0.130
Postmenopausal	167 (34)	233 (40.3)	
Undetermined	94 (19.1)	14 (2.4)	
Specimen	491	598	
Biopsy	118 (24)	373 (62.4)	
Breast lumpectomy	257 (52.4)	12 (2)	
Mastectomy	116 (23.6)	213 (35.6)	
Tumor size (cm)	299	299	
Median	2.5	2.8	0.252
<2 cm	87 (29.1)	74 (24.7)	
≥2 cm	212 (70.9)	225 (75.3)	
Histological subtype	481	586	
IC NOS*	440 (91.5)	519 (88.6)	
ILC**	21 (4.4)	40 (6.8)	
Others	20 (4.1)	27 (4.6)	
Histological grade	462	555	0.659
I	51 (11)	35 (6.3)	
II	203 (44)	293 (52.8)	
III	208 (45)	227 (40.9)	
Lymph node status	223	204	0.926
N0	79 (35.4)	84 (41.2)	
N+	144 (64.6)	120 (58.8)	
Estrogen receptor	385	571	0.186
Positive	311 (80.8)	486 (85.1)	
Negative	74 (19.2)	85 (14.9)	
Progesteron receptor	378	499	0.301
Positive	266 (70.4)	321 (64.3)	
Negative	112 (29.6)	178 (35.7)	
HER2	283	385	0.123
Negative	197 (69.6)	325 (84.4)	
Positive	86 (30.4)	60 (15.6)	
Ki‐67	491	544	0.426
<20%	85 (17.3)	137 (25.2)	
≥20%	406 (82.7)	407 (74.8)	
Immunohistochemical classification	268	263	0.673
Luminal A	156 (58.3)	181 (68.8)	
Luminal B	59 (22)	22 (8.4)	
HER2 positive	25 (9.3)	18 (6.8)	
Triple negative	28 (10.4)	42 (16)	

NOS *= Not Otherwise Specified; ILC **= Invasive lobular carcinoma.

Comparing Charles Nicolle, Jendouba and Military hospitals' series, median ages were 50, 53 and 48 years, respectively. Tumor size was ≥2 cm in 69/86 (80.2%) cases, 90/117 (77%) cases, and 53/96 (55.2%) cases in Charles Nicolle, Jendouba and Military hospitals, respectively. In the three Tunisian hospitals, luminal A was the predominant subtype found in 35/80 (43.7%) cases in Charles Nicolle, 77/110 (70%) cases in Jendouba and in 44/78 (56.4%) cases in Military hospitals. Clinical and pathological characteristics of BCs in the Tunisian series are summarized in Table 2.

**TABLE 2:** Clinical and tumor characteristics of breast carcinomas among Tunisian hospitals.

Variables	Charles Nicolle hospital, *n* (%)	Jendouba hospital, *n* (%)	Military hospital, *n* (%)
Total, *n*	127	211	153
Age			
Range (years)			
Median	50	53	48
Age category			
<30	5 (4)	4 (1.9)	3 (2)
31–40	13 (10.1)	28 (13.3)	34 (22.2)
41–50	48 (37.8)	54 (25.6)	60 (39.2)
51–60	38 (30)	55 (26)	26 (17)
61–70	13 (10.2)	40 (19)	14 (9.1)
>70	10 (7.9)	30 (14.2)	16 (10.5)
Specimen	127	211	153
Biopsy	30 (23.7)	60 (28.4)	28 (18.3)
Breast lumpectomy	73 (57.4)	80 (38)	104 (68)
Mastectomy	24 (18.9)	71 (33.6)	21 (13.7)
Tumor size (cm)	86	117	96
Median	3	2	2
<2 cm	17 (19.8)	27 (23)	43 (44.8)
≥2 cm	69 (80.2)	90 (77)	53 (55.2)
Histological subtype	124	206	151
IC NOS*	107 (86.3)	193 (93.7)	140 (92.7)
ILC**	6 (4.8)	9 (4.4)	6 (4)
Others	11 (8.9)	4 (1.9)	5 (3.3)
Histological grade	120	199	143
I	21 (17.5)	14 (7)	16 (11.2)
II	56 (46.7)	84 (42.2)	63 (44)
III	43 (35.8)	101 (50.7)	64 (44.8)
Lymph node status	50	103	50
N0	19 (38)	45 (43.7)	15 (30)
N+	31 (62)	58 (56.3)	35 (70)
Estrogen receptor	90	173	122
Positive	85 (94.4)	129 (74.6)	97 (79.5)
Negative	5 (5.6)	44 (25.4)	25 (20.5)
Progesteron receptor	80	181	117
Positive	36 (45)	143 (79)	87 (74.4)
Negative	44 (55)	38 (21)	30 (25.6)
HER2	90	108	85
Negative	42 (46.7)	83 (76.9)	72 (84.7)
Positive	48 (53.3)	25 (23.1)	13 (15.3)
Ki‐67	127	211	153
<20%	30 (23.6)	34 (16.1)	21 (13.7)
≥20%	97 (76.4)	177 (83.9)	132 (86.3)
Immunohistochemical classification	80	110	78
Luminal A	35 (43.7)	77 (70)	44 (56.4)
Luminal B	20 (25)	20 (18.2)	19 (24.4)
HER2 positive	10 (12.5)	5 (4.5)	10 (12.8)
Triple negative	15 (18.8)	8 (7.3)	5 (6.4)

NOS *= Not Otherwise Specified; ILC **= Invasive lobular carcinoma.

## 4 DISCUSSION

The present study is to our knowledge the first published one comparing the clinical and histopathological characteristics of BC in Tunisia and Algeria. BCs in the two countries share almost the same clinical and pathological characteristics: A young age, large tumor size and high rate of axillary lymph node tumor involvement at diagnosis.

The highest frequency of BC was observed in the age group of 41 to 50 years, with a median age of 50 and 48 years in the Tunisian and Algerian series, respectively. These results are consistent with the findings of previous Tunisian series which reported an average age of 50 years with a peak frequency between 41 and 50 years.^22^, ^23^, ^24^ Similarly, in previous Algerian studies, median age at diagnosis was 47 years. In another Algerian series, two peak incidences were described: One at the fifth decade and the other in the age group of 70–74 years.^25^, ^26^ A comprehensive literature review of reports of BC in Arab countries reported a median age of 44.5 years.^27^ BCs in Tunisia and Algeria occur at an earlier age compared to BCs in Western countries in which peak incidence of BC is between 60 and 70 years.^28^ Young age of BC in Africa is probably due to young population and genetic and environmental factors.^27^, ^29^

In the present study, median tumor size was 2.5 and 2.8 cm in the Tunisian and Algerian series, respectively. Tumor size in Tunisia declined from 4.9 cm in 1991^30^ to 3 cm in 2017^31^ and 2.5 cm in our series but is still greater than BC's size in Western countries which is reduced to 1.5 cm under the effect of mass screening and early detection campaigns.^32^ In Algeria, a recent study reported a mean tumor size of 3.6 cm.^33^ In Algeria and Tunisia, BC mainly concerns a young population, thus mammographic screening should start at the age of 40.

In addition to high tumor size, BCs in the two series of the present study were characterized by a high frequency of axillary lymph node metastases at diagnosis (64.6% and 58.8%). Similar frequencies were reported in other African countries like Morocco (60%),^34^ Lybia (73.9%)^35^ and Nigeria (79%).^36^ These frequencies are higher to those reported in Europe (34%).^37^ In Tunisia and Algeria, women still consult tardily with palpable lesions. Mammography is hardly accessible in some regions due to its high cost and inequalities in territorial imaging centers' distribution. Training of radiologists to ensure quality, validity and interpretation criteria deserve to be deployed to popularize mammography for screening purposes. In the meantime, prevention strategies should raise awareness by promoting self‐examination and systematic clinical examination of women's breast.^38^

The frequency of BC categories subtypes is highly variable. Luminal A BC was the predominant category in Tunisian and Algerian series (58.3% and 68.8%, respectively). The predominance of luminal A subtype was also reported in previous studies in Tunisian,^16^, ^20^ Algerian,^39^ and Western series.^11^ Lowest frequencies of luminal A were reported in other African countries such as Mali (29.2%)^40^ and Ghana (25.6%).^41^ In this study, the frequency of luminal B, in the Algerian series, is relatively low (8.4%) compared to Tunisian series (22%) and to a previous Algerian one which reported a rate of 19.7% of luminal B BCs.^39^ The rates of luminal B BCs in our Tunisian series were concordant to those of previous Tunisian series.^5^, ^26^ Other studies, like a recent Tunisian one and others from Saudi Arabia and Italy found a high rate of luminal B BC.^42^, ^43^, ^44^ Although the molecular classification is well codified, there are some technical biases such as specimen fixation, sample storage duration, and other preanalytical immunohistochemical variables which could influence the accuracy of IHC results.

In Western countries, the prevalence of HER2 positive BCs varies from 4%^45^ to 21.6%.^46^ In this study, HER2+ BCs were observed in 10.4% and 6.8% in the Tunisian and Algerian series, respectively.

In this study, triple negative BCs were found in 10.5% in the Tunisian series. Previous Tunisian studies reported variable triple negative BCs' frequencies: 22.5%,^20^ 15.5%,^42^ and 14%.^47^ In the Algerian series of this study, the rate of triple negative BCs was 13.7%, similar to the Tunisian one, but lower than that described in another Algerian series (20.8%).^39^ Rates of triple negative BCs in both series of this study are similar to those of European countries.^48^ In the literature, rates of triple negative BCs vary from 10% to 25%.^47^ High prevalence of triple negative BCs are reported in Senegal and Nigeria reaching 55%.^49^ High rates of triple negative BCs among Sub‐Saharan African women are not well understood. Some authors suggested that these differences could be partly explained by genetic influences, ethnicity and race factors.^52^, ^53^ Differences in rates of BC categories may be related also to variations in immunohistochemistry techniques and interpretation. The negativity of hormone receptors and HER2 suggests challenges with tissue collection and processing, which leads to inaccurate IHC results. Necrotic tumor specimens, a prolonged delay before fixation, doubtful quality fixation, a prolonged stay in fixative, inadequate laboratory techniques, and insufficient quality assurance/quality control procedures are just a few of the issues.

Categories of BCs have been defined based on techniques such as immunohistochemistry and fluorescent in situ hybridization.^50^ Recently, technological breakthroughs in sequencing have paved the path for the use of next‐generation sequencing (NGS). In BC, NGS has several documented applications in a variety of clinical settings. One of the applications of NGS is inherited cancer testing by targeted NGS panels focusing on high‐penetrant/high‐risk mutated genes, such as BRCA1 and BRCA2. Another key clinical application of NGS is the detection of driver mutations in BCs.^51^ In Tunisia and Algeria, the use of NGS is not applied in routine. It remains only in the framework of research of germline BRCA mutations.^52^, ^53^

This study has some limitations. Menopausal status, comorbidities, family history of cancer, metastatic stage, treatment and follow‐up outcomes were not examined due to unavailability of data. The categorization of triple‐negative cases into subgroups was not possible due to missing data for other biomarkers like cytokeratin 5/6, cytokeratin 14, and EGFR. The lack of gene expression profiling in our study did not allow to determine the prevalence of molecular BC subtypes with great precision. Despite the stated limitations, this study is the first to compare clinicopathological particularities of BC among Tunisian and Algerian patients, adding strength that there are no disparities between the two North African countries and that BC is characterized by poor prognostic factors: young age, large tumor size and high rate of lymph nodes involvement at diagnosis. Therefore, priority should be given to implementing BC's awareness programs, which have been shown to raise cancer awareness knowledge and thereby reduce preventable deaths from BC. This should be addressed as soon as possible by establishing cancer registries before the burden of BC and other chronic diseases drastically increases due to the epidemiological change that has already begun in Africa.

## 5 CONCLUSION

This study demonstrated that BCs in Tunisia and Algeria share almost the same characteristics concerning young age, tumor size, and high rate of lymph nodes involvement at diagnosis. Promoting BC screening, raising awareness of women to self‐examination as well as midwives and doctors to systematic breast examination during consultations is mandatory to detect BCs at early stages.

## AUTHOR CONTRIBUTIONS

**Farah Sassi**: Guarantor of integrity of the entire study, Study concepts and design, Literature research, Statistical analysis, Manuscript preparation and Manuscript editing. **Meriem Ben Rekaya**: Study concepts and design, Data analysis, Manuscript preparation and Literature research. **Ayed Belarbi**: Guarantor of integrity of the entire study, Study concepts and design and Manuscript preparation. **Dalia Chilla**: Clinical studies. **Nada Mansouri**: Clinical studies and Data collection. **Leila Achouri**: Clinical studies and Data collection. **Essia Saied**: Data collection. **Reda Kassa**: Clinical studies. **Linda Belhaj Kacem**: Literature research. **Manel Ouezani**: Literature research. **Nadjiba Debabeche**: Clinical studies. **Fatima Rebhi**: Data collection. **Soumaya Rammeh**: Guarantor of integrity of the entire study, Study concepts and design and Manuscript preparation. All authors read and approved the final version of this manuscript.

## CONFLICT OF INTEREST STATEMENT

The authors have stated explicitly that there are no conflicts of interest in connection with this article.

## ETHICS STATEMENT

The ethical approval was obtained by the Ethical Research Committee of Charles Nicolle hospital. The study was carried out as part of the Tunisian‐Algerian Research Project entitled: “Study of tumor biomarkers for theranostic purposes with the aim of setting up validated molecular tests in clinical practice”: PRD/TN/DZ/21/08.

## Data Availability

The datasets used and/or analysed during the current study are available from the corresponding author on reasonable request.
